# Mission Availability for Bounded-Cumulative-Downtime System

**DOI:** 10.1371/journal.pone.0065375

**Published:** 2013-07-02

**Authors:** Yu Zhou, Gang Kou, Daji Ergu, Yi Peng

**Affiliations:** 1 School of Management and Economics, University of Electronic Science and Technology of China, Chengdu, China; 2 School of Business Administration, Southwestern University of Finance and Economics, Chengdu, China; 3 Southwest University for Nationalities, Chengdu, China; University of Adelaide, Australia

## Abstract

In this research, a mathematics model is proposed to describe the mission availability for bounded-cumulative-downtime system. In the proposed model, the cumulative downtime and cumulative uptime are considered as constraints simultaneously. The mission availability can be defined as the probability that all repairs do not exceed the bounded cumulative downtime constraint of such system before the cumulative uptime has accrued. There are two mutually exclusive cases associated with the probability. One case is the system has not failed, where the probability can be described by system reliability. The other case is the system has failed and the cumulative downtime does not exceed the constraint before the cumulative uptime has accrued. The mathematic description of the probability under the second case is very complex. And the cumulative downtime in a mission can be set as a random variable, whose cumulative distribution means the probability that the failure system can be restored to the operating state. Giving the dependence in the scheduled mission, a mission availability model with closed form expression under this assumption is proposed. Numerical simulations are presented to illustrate the effectiveness of the proposed model. The results indicate that the relative errors are acceptable and the proposed model is effective. Furthermore, three important applications of the proposed mission availability model are discussed.

## Introduction

### Background and motivation

Assuming a repairable system performs one mission type in the same operational environment, the system will be repaired immediately on an operational failure. During the mission time, short downtime could be tolerated. But the cumulative downtime cannot exceed a bounded cumulative downtime before the cumulative uptime has accrued. If the cumulative downtime exceeds the bounded cumulative downtime, not only the mission will fail, but also a penalty will be paid. This kind of system can be found in the nuclear and food industries, and a special system of this kind was examined by Gupta et al. [1]. We always pay close attention to two common dependability measures - reliability and availability. Reliability, defined as the probability that the system remains operational over an observation period, is an appropriate measure for evaluating the effectiveness of systems where no down time can be tolerated [2], [3]. Availability, defined as the probability that the system is operating satisfactorily at any point in time under stated conditions, is a more appropriate measure for systems which are usually operated continuously and short down times can be tolerated during their operation [3]–[5]. The choice of a dependability measure often requires a trade-off between the two common dependability measures [6]–[11].

Although there are many categories on availability based on the different definitions of uptime and downtime in the literature, such as interval availability [12], achieve availability [13], and steady-state availability [14], detailed overview of availability can be found in [5]. Availability application can also be found in production planning, maintenance scheduling and so on [9]–[14]. However, for the bounded-cumulative downtime system, the cumulative downtime and cumulative uptime must be considered simultaneously. The existing availability model could not describe the availability characteristics exactly. It is important to set up a model for availability analysis of the bounded-cumulative downtime system.

### Literature overview

Through the previous literatures, we found some early literatures introducing the availability analysis of the bounded-cumulative downtime system. And the most appropriate description should be mission availability [15]. Corresponding to the failure constraint, the mission availability can be described as the probability that the downtime (cumulative downtime or cumulative failure number in a mission) does not exceed the bounded downtime (bounded cumulative downtime or bounded cumulative failure number) constraint before the total operating time has accrued [1], [16]. Although it is important in applications where system bounded downtime can be tolerated [16], [17], the mission availability is less studied.

For the bounded downtime, Birolini [14] proposed a closed form expression of the mission availability for a system modeled by an alternating renewal process. Birolini calculated the mission availability by summarizing all the possibilities of having *n* failures (*n* = 0, 1, 2 …) during the total operating time. Csenki [15] modeled the mission availability in a semi-Markov process. A closed form solution was also derived. However, as Csenki admitted, this solution is not suitable for computational work. Kodama et al. [18] analyzed system mission reliability for a one-unit system with allowed downtime. Gupta et al. [16], [17] discussed a two-unit cold standby system where each unit can work in three modes and bounded downtime. They analyzed the system by utilizing regeneration points and discussed mean time to system failure, point availability and steady-state availability besides mission availability. Similarly, Dunbar [19] presented an expression for the probability of a failure of a system consisting of two components. When a system is declared to be failed, both components must fail and remain in the failed state for at least a given finite time. However, the total operating time is a constant. Furthermore, the cumulative operating time is random because of the random failure numbers and downtime of each individual failure.

For the constraint of cumulative downtime, Birolini [11] also proposed a closed form expression of the mission availability. Gao and Zhu [17] proposed a simulation algorithm of cluster system mission availability. Nicola and Bobbio [18] discussed unified performance and reliability analysis of a system which alternates between up state and down state. The system could reach a catastrophic condition when the cumulative downtime exceeds a critical threshold. A mission will be completed with a specified amount of work before the system reaches the critical threshold. The preemptive-resume and preemptive-repeat failure were considered respectively. Based on Markov for unified performance measures, closed-form expressions, such as system lifetime, mission reliability, interval availability and instantaneous availability, have been obtained. However, the mission availability has not been considered. In this case, the total operating time is defined as a constant again. Furthermore, the cumulative operating time does not have any constraint since the failure numbers and failure time of each individual failure are random. Although the cumulative operating time was constrained in Gao et al. [17] and Nicola et al. [18], the closed form expression of mission availability has not been given out.

Goyal and Nicola et al. [20] discussed the constraint of bounded number of downtimes. In their study, the preemptive-resume failure and preemptive-repeat failure were also considered. However, only the expressions of system lifetime and the probability of mission completion were specified.

### Objective and outline

In the existing studies, the alternating renewal process and simulation method are widely used to analyze the mission availability. Besides, the total operating time and the cumulative downtime were seldom considered as constraints simultaneously. To the best of our knowledge, only the researchers in Gao et al. [21] and Nicola et al. [22] considered the total operating time and the cumulative downtime as constraints simultaneously. However, the closed form expression of mission availability was not given out. As an attempt, we will take all the failures as one failure during a mission, and the cumulative downtime of assumed failure is equal to the sum of all the failure downtime. In addition, we will process the cumulative downtime and cumulative uptime as constrains simultaneously. So the mission availability can be described as the probability that the cumulative downtime does not exceed the bounded cumulative downtime before the cumulative uptime has accrued. The probability should include two mutually exclusive cases. One case is the system has not failed, where the probability can be described by system reliability. The other case is the system has failed and the cumulative downtime does not exceed the constraint before the cumulative uptime has accrued. In the present study, we will try to use the cumulative downtime distribution of the assumed failure to describe the probability. A mission availability model under this assumption is proposed, giving the dependence in the scheduled mission. Numerical examples are presented to illustrate the effectiveness of the proposed model. Three important applications of the proposed mission availability model, such as design and optimal analysis, mission scheduling, are discussed.

Comparing with the existing literatures, the differences between our study and the existing researches are reflected in the followings:

1. Unlike literatures [14]–[18], the cumulative uptime and the cumulative downtime are considered as constraints simultaneously in our study.
2. The proposed mission availability model has closed form expression. Although Gao et al. [20] and Nicola et al.[18] considered the cumulative uptime and the cumulative downtime as constraints simultaneously, the closed form expression of mission availability was not given out.
3. Numerical simulations are presented to illustrate the effectiveness of the proposed model.

The rest of the paper is organized as follows. The next section formulates the problem and gives out the assumptions. Section 3 develops the mission availability model. Numerical simulations are presented in Section 4. Model applications are discussed in Section 5. Limitations of the study, open questions, and future work are discussed in Section 6, and conclusions are in Section 7.

## Problem Formulation and Assumptions

### Problem formulation

**Figure 1:**
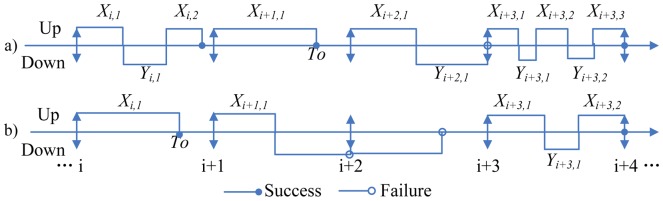
Execution process of system missions.

Considering a system performs one mission type in the same operational environment, the system will be repaired immediately upon an operational failure. System executing a mission successfully must work cumulative 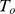
units of time. Meanwhile, the cumulative downtime constraint must be satisfied. In a word, the cumulative downtime cannot exceed the bounded cumulative downtime before the cumulative uptime 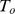
has accrued. The execution process of system missions is displayed in Figure 1. In Figure 1, 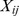 denotes the uptime between failures during the 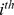 mission. 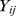 denotes the downtime of 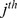 failure during the 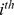 mission. Let 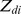 denotes the cumulative downtime during the 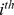 mission. It is equal to the sum of all the failures downtime during the 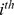 mission, and calculated as: 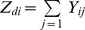.

### Assumptions

In extant literatures, we found that it is very difficult to model 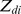 when carrying out mission availability analysis. The alternating renewal process and simulation method are widely used to model or simulate 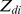. In the present paper, we will use an approximate method to solve this problem. Unlike the alternating renewal process and simulation method, we assume 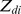 as a random variables. The approximate distribution of 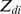 can be determined by statistical method. In another words, we assume that the approximate distribution of 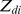 during the 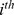 mission can describe the failure behavior. Besides, there are some assumptions as follows.

1. System downtime contains the direct repair time and indirect waiting time (e.g. the time spent on failure detecting, failure diagnosis and preparing the spare parts) [23], [24].
2. We do not care the differences among the component failures.
3. Assume the repair is perfect and the system can be restored to the state as new [25].
4. Under the assumptions (2) and (3), 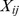, 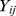 and 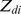 are independent and identically distributed [26]. So we set the cumulative failure distribution (CDF for short) of 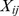, the cumulative failure distribution of 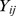 and cumulative repair distribution (CRF for short) of 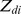 as 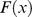, 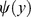 and 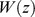, respectively. Such that, 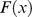 represents the probability that the system will fail before its cumulative uptime reaches 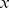. 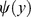 represents the probability that the system can be resorted to the state as new before the downtime reaches 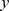. While 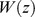 represents the probability that the system can be resorted to the state as new before the cumulative downtime reaches 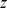.
5. System's 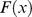 and 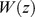 can be obtained through fitting the field failure and repair data.
6. *T*, 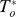, 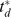 are determined by operation manager or optimization.

## Model Development

**Figure 2:**
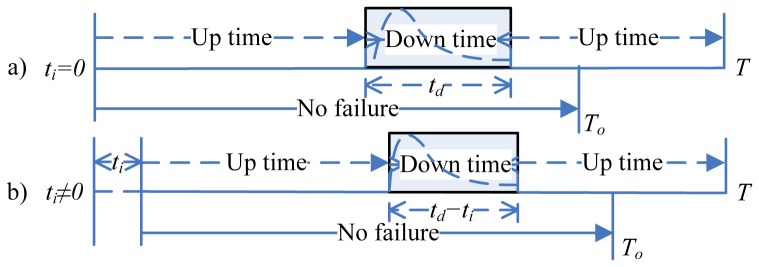
Schematic figure of the proposed mission availability model.

According to the background and assumptions mentioned above, we can describe the proposed mission availability model as shown in Figure 2. So mission availability can be delimited as the probability that system cumulative downtime does not exceed 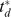 before the cumulative uptime 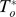
has accrued. Due to the failure occurred in the last mission may delay the start of the following *n* missions, the delay-start time can be decomposed in two scenarios [27]:

Scenario 1: the first scenario happens when the following missions are not delayed, which means the system is in the operating state all the time when the next mission starts.

Scenario 2: the second scenario happens when the following missions may be delayed. We stated them as mission independence and mission dependence as shown in Figure 1 a) and b) respectively. Set the delay-start time of the 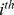 mission as 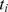. So, 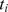 = 0 means the scheduled mission is independent. Otherwise, the scheduled mission is dependent.

Next, the mathematical description will be given out.

### Scenario 1

Mission independence means the system is in the operating state all the time when the mission starts. Therefore, the mission availability denoted as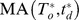 can be defined as [14]:
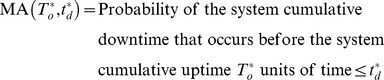
(1)


In the equation (1), two mutually exclusive cases should be considered that the system can execute a mission successfully:

Case 1: the system operates 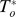 units of time without a failure;

Case 2: the system encounters failure, while the cumulative downtime cannot exceed the bounded cumulative downtime before the cumulative uptime 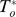 has accrued.

For the case 1, the system has not failed during 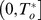 with probability .

For the case 2, the system has failed, but system cumulative downtime does not exceed the bounded cumulative downtime before the cumulative uptime has accrued. According to the assumption 2), if there is a failure during the 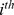
mission, the system must fail in the time interval 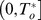 with the probability 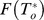. The probability that the system can be restored to the state as good as new within cumulative downtime 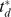 is 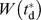 So the probability that the system has failed but system cumulative downtime does not exceed the bounded cumulative downtime before the cumulative uptime has accrued, is 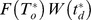.

Thus, the mission availability 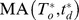 can be expressed as:
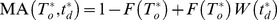
(2)


### Scenario 2

For the scenario of mission dependence, the following 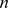 missions may be delayed because of the long downtime during the 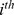
mission. Therefore, the probability that scheduled mission start successfully must be considered in two mutually exclusive cases:

Case 1: When 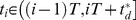, the scheduled mission can start, but the bounded cumulative downtime has reduced to 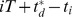. The probability that system cumulative downtime does not exceed 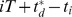 before the cumulative uptime 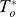 has accrued can be denoted as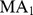.
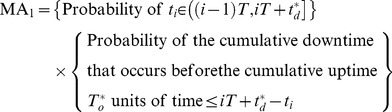
(3)


Let 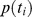 denotes the probability that 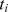 can get any value in 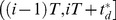. For the discrete random variable 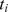, let 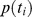 denotes the probability that 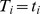. 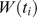 is the probability that 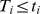. The relations between 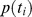 and 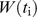 are given by:
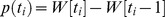
So the probability 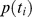 can get any value in 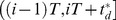 is:
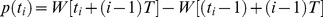
(4)Following equation (2), the probability that system cumulative downtime does not exceed 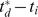 before the cumulative uptime 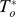 has accrued can be represented as:
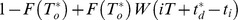
If 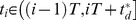
and system cumulative downtime does not exceed 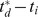 before the cumulative uptime 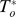 has accrued, then the probability can be computed as:
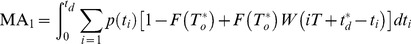
(5)


Case 2: the system has not failed during the last mission or the system can be restored to the state as good as new before the next mission starts. The probability that system cumulative downtime does not exceed 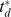 before the cumulative uptime 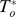 has accrued is denoted as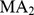, and calculated by:
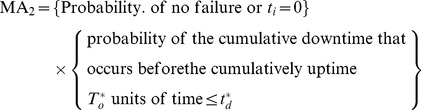
(6)The probability with no failure is 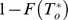. And the probability that the 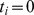 can be represented as:
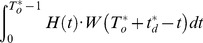
where 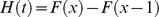. So, the probability when no failure or 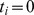 is:
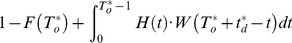
(7)According to the equations (2) and (6), we can get:
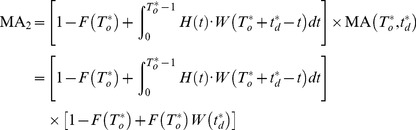
(8)


Adding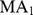 to 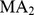, the mission availability 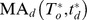 under the case of mission dependence becomes:
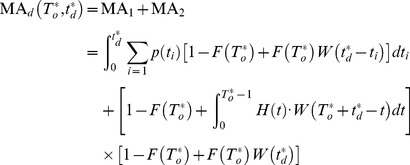
(9)


Until now, the mission availability model has been proposed. The modeling process will be further discussed as follows.

### Parameter estimation and goodness-of-fit test

According to the equations (2) and (9), the critical modeling step of the mission availability is to determine 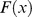 and 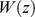. In the practice, 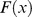 and 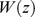 can be obtained through fitting the field failure and repair data by the common models, such as Weibull, Exponential, Gamma and Lognormal model. The Maximum Likelihood method can be used to estimate the parameters. Take Weibull model as example, the likelihood function can be presented as:
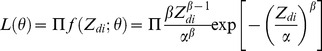
which is also given by:
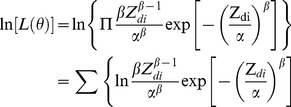
(10)where 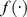 is the probability density function of the fitted model. The model parameters can be obtained by directly maximizing.

The Chi-Squared Test can be used to test the goodness-of-fit, which is given by Blischke and Murthy [2]:
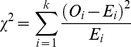
(11)Take Weibull model as example, 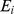 can be presented as:
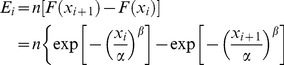
(12)where 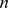 is the sample size, with each observation falling into one of 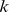 possible classes (A rule of determining 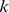 is that the expected frequency 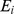 should satisfy 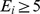. Otherwise, to combine classes if 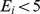.). 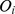
is the observed frequency in class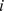, and 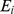 is the expected frequency. The smaller the 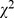 is, the better the fitted model is.

Thus, once the model parameters of 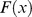 and 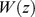 are determined, the mission availability can be calculated. Numerical simulations will be presented to verify the rationality of the assumption and the effectiveness of the proposed model next.

## Numerical Examples

Monte Carlo (MC for short) simulation is a commonly used simulation method [28]. The MC simulation is thus adopted in our numerical simulations. According to the assumption and the proposed mission availability model, the rationality of obtaining 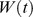 is the key assumption. So we pay more attention to this assumption in the simulation examples, and four common models are used to test the cumulative repair distribution. The simulation frame can be summarized as follows.

**Figure 3:**
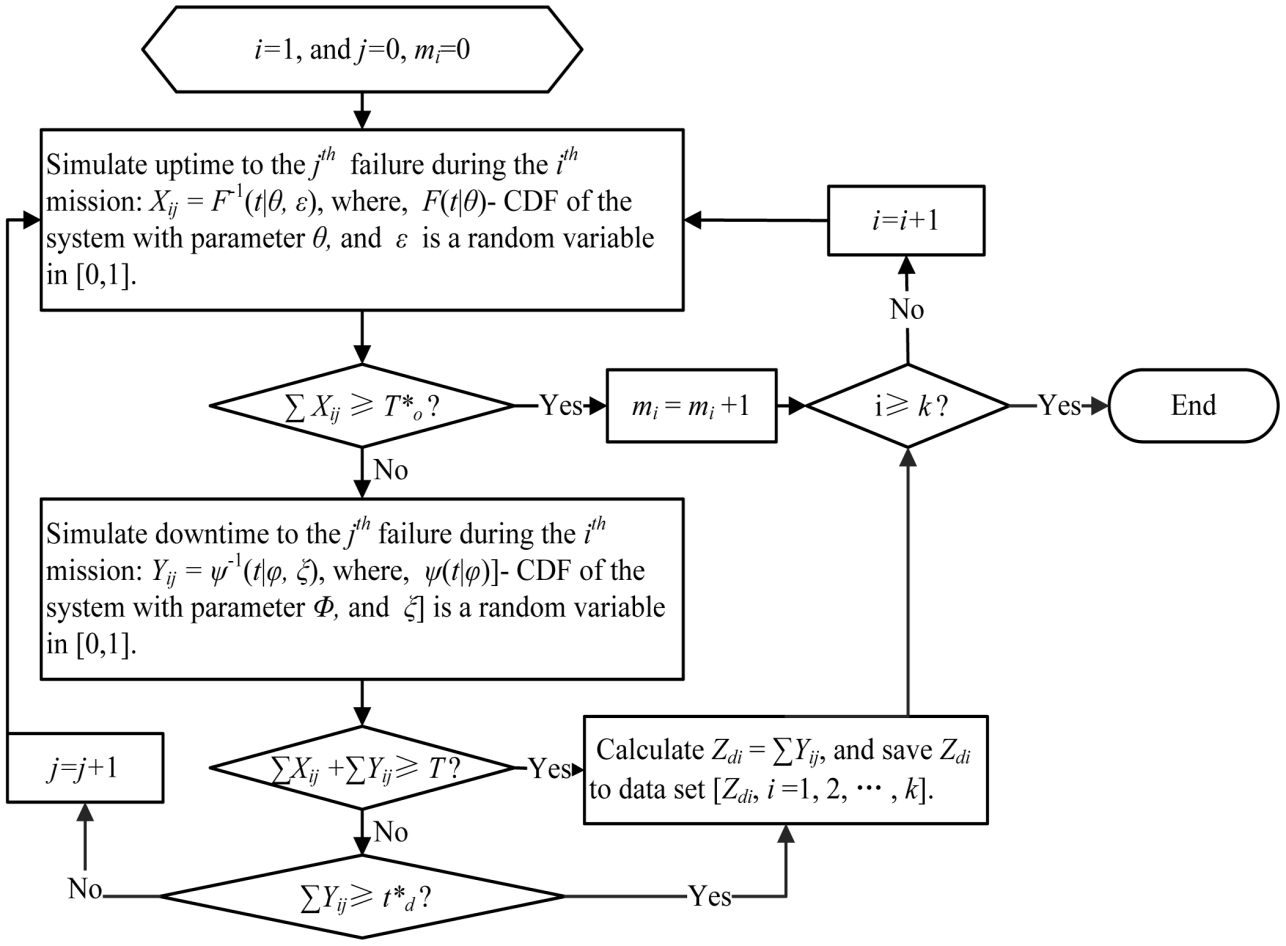
MC simulation procedure of mission independence.

**Figure 4:**
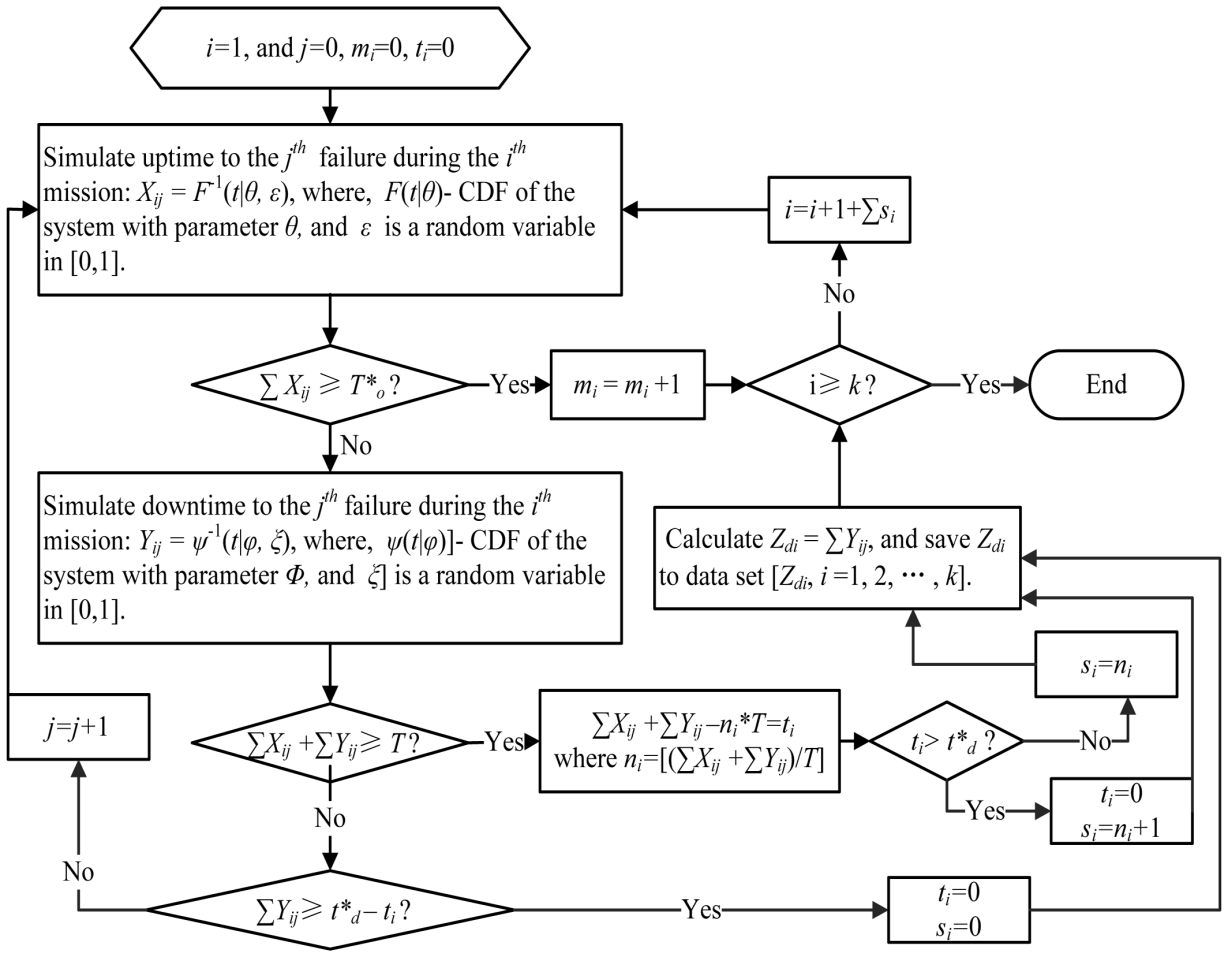
MC simulation procedure of mission dependence.

1. Define *T*, 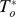, 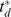, number of simulation times *k*, where *T*, 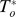, 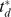 are constants (this can be determined by operation manager or optimization).
2. Using the MC simulation to simulate the execution process of system missions as shown in Figure 1 with given 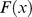 and 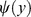. The detailed MC simulation procedure of mission independence and dependence are displayed in Figure 3 and Figure 4 respectively.
3. Calculating the observed mission availability MA = *m_k_*/*k* and export the data set [*Z_di_*, *i* = 1, 2, …, *k*]. Needing to clarify, [*Z_di_*, *i = i*+1,…,*s_i_*] will be null if the following 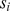 missions is delayed because of the long downtime during the 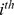
mission.
4. Fit the downtime data set [*Z_di_*, *i* = 1, 2, …, *k*] using the common distributions (weibull, lognormal, gamma, exponential, normal, and so on), through the goodness-of-fit finding the best model *W*(*t*).
5. Calculating mission availability with equations (2) or (9) and the relative errors (RE): RE = 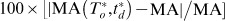 or RE = 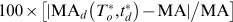.

In order to ensure the credibility of the simulation examples, the followings are considered in setting variables.

1. Different combinations of 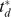
and 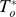.
2. Different cumulative repair distribution type of 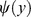.
3. Different distribution parameters of 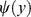.

The detailed simulation variables are listed in Table 1.

**Table 1:** Simulation variables and results.

t_d_/T_o_	Simulation model of Y_ij_ (F(T_o_) is Exponential distribution with failure rate 0.006)					
	Exponential	MRE-I (%)	MRE-D (%)	Lognormal	MRE-I (%)	MRE-D (%)
0.20	0.02	1.10	0.71	(3.00,0.90)	0.67	0.32
	0.06	1.26	0.59	(2.00,0.90)	0.36	0.26
	0.10	0.92	0.43	(1.00,0.90)	0.24	0.22
	0.14	0.44	0.35	(2.00,0.50)	0.73	0.35
	0.18	0.13	0.29	(2.00,1.30)	0.99	0.87
0.30	0.02	1.46	0.55	(3.00,0.90)	1.23	0.87
	0.06	1.15	0.46	(2.00,0.90)	0.26	0.29
	0.10	0.41	0.51	(1.00,0.90)	0.21	0.30
	0.14	0.12	0.31	(2.00,0.50)	0.05	0.18
	0.18	0.10	0.38	(2.00,1.30)	0.42	0.33
0.40	0.02	1.35	0.67	(3.00,0.90)	0.66	0.72
	0.06	0.69	0.56	(2.00,0.90)	0.20	0.45
	0.10	0.09	0.36	(1.00,0.90)	0.10	0.16
	0.14	0.04	0.40	(2.00,0.50)	0.10	0.43
	0.18	0.03	0.32	(2.00,1.30)	0.21	0.23

	Gamma	MRE-I (%)	MRE-D (%)	Weibull	MRE-I (%)	MRE-D (%)
0.20	(6.00,1.50)	0.25	0.24	(10.00,1.20)	0.74	0.65
	(10.00,1.50)	1.10	0.44	(15.00,1.20)	0.78	1.03
	(14.00,1.50)	0.96	0.56	(20.00,1.20)	1.14	0.93
	(10.00,0.50)	0.02	0.08	(10.00,0.80)	0.21	0.46
	(10.00,2.50)	0.88	0.43	(10.00,1.60)	0.19	0.11
0.30	(6.00,1.50)	0.19	0.21	(10.00,1.20)	0.45	0.48
	(10.00,1.50)	0.71	0.37	(15.00,1.20)	0.75	0.43
	(14.00,1.50)	0.95	0.72	(20.00,1.20)	1.03	1.00
	(10.00,0.50)	0.13	0.09	(10.00,0.80)	0.12	0.28
	(10.00,2.50)	0.74	0.54	(10.00,1.60)	0.05	0.27
0.40	(6.00,1.50)	0.07	0.04	(10.00,1.20)	0.15	0.73
	(10.00,1.50)	0.40	0.55	(15.00,1.20)	0.54	0.58
	(14.00,1.50)	0.94	0.67	(20.00,1.20)	0.83	0.62
	(10.00,0.50)	0.07	0.06	(10.00,0.80)	0.06	0.33
	(10.00,2.50)	0.59	0.43	(10.00,1.60)	0.05	0.27

MRE is mean relative error. I and D denote independence and dependence.

Set the simulation times, denoted as *k* in Figure 3, to be 50000. Then we can get the simulative mission availability values and the downtime data set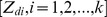. The common models are used to fit the downtime data set. The model parameters are estimated by the Maximum Likelihood method whilst the goodness-of-fit can also be obtained. Through the Chi-Squared test, the appropriate model can be determined. Then, the mission availability values with the proposed mission availability model and the relative errors are calculated. The mean relative errors are displayed in Table 1. The simulation results show that almost all the mean relative errors of each simulation are less than 1.5%. Generally speaking, this relative error can be accepted [29]. As is shown in the mission availability estimation, the maximum percent error is 1.46. This implies that there is no noticeable impact on the actual mission availability. So the assumption, which takes the cumulative downtime in a mission as a variable to model the mission availability, is rational and the proposed model is effective. Studying the proposed mission availability model, the estimated error may come from the fitted 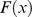 and 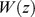. If more accurate results are expected, more attention should be paid to fit more accurate model of 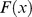 and 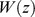 in the future research.

## Model Applications

In this section, three important applications of the proposed mission availability model will be discussed. Firstly, it can be used to carry out mission availability analysis. The relationship among reliability, maintainability and mission availability can be obtained. Secondly, this model can be used in system design and optimum analysis. The optimal levels or not-saturation interval of reliability and maintainability can be determined when the mission availability is set as a requirement value. Finally, a cost function will be given out to determine the optimal bounded cumulative downtime in mission scheduling. In addition, the proposed model may have some other potential applications. For example, reliability and maintainability allocation, determining the improvement indirect and target at the reliability improvement, maintenance resources optimization and scheduled maintenance. These potential applications will be further researched in the future work.

### Mission availability analysis

**Figure 5:**
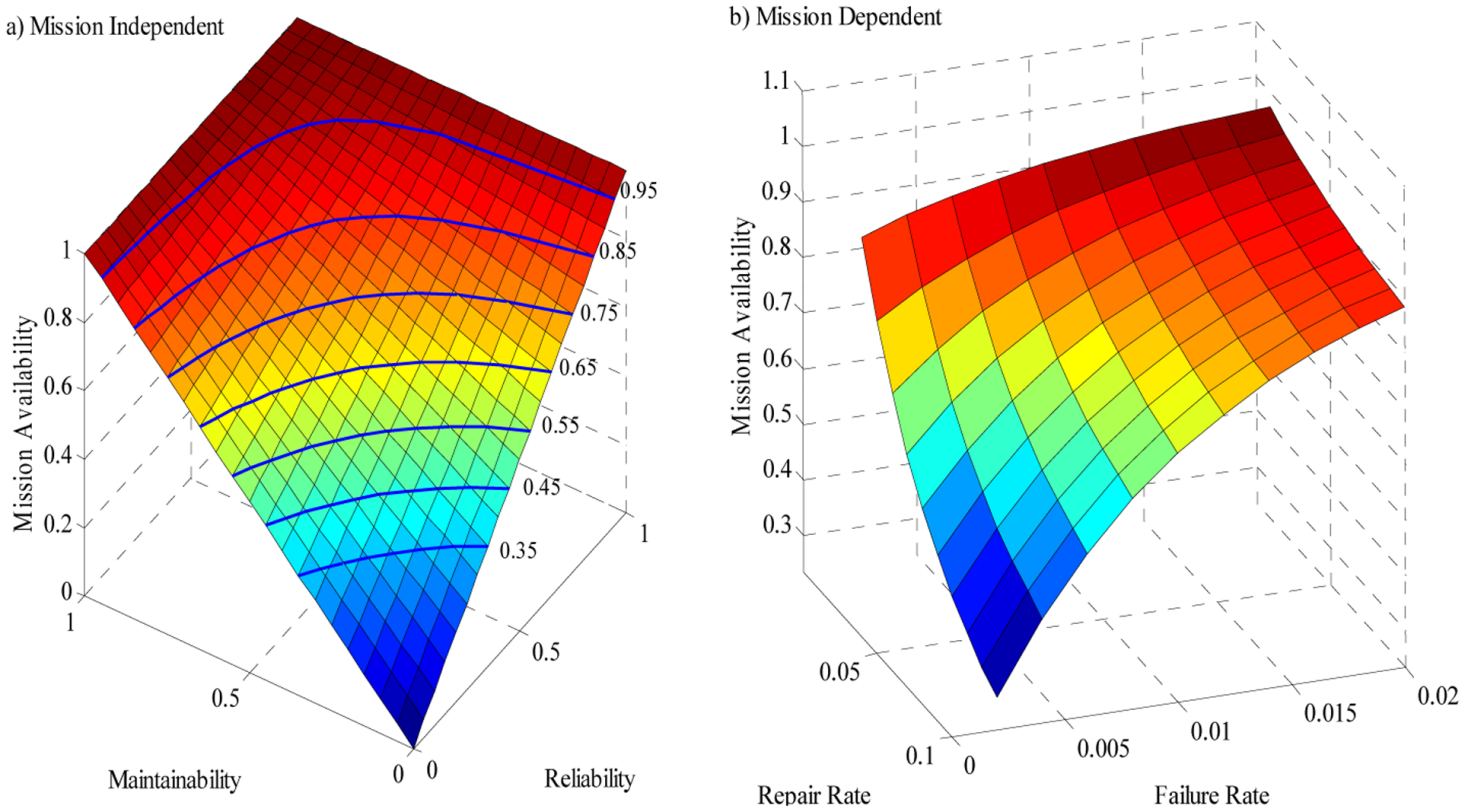
Relationship among mission availability, reliability and maintainability. (a) Scenario 1: Mission independence. The highlighted curves are the equal-availability curves with the availability values 0.35(0.1)0.95. (b) Scenario 2: Mission dependence. With exponential reliability rate 0.01(0.01)0.1 and Exponential repair rate 0.002(0.002)0.02.

Mission availability contains reliability and maintainability characteristics. Through mission availability analysis, the relationship among them can be obtained. For the mission availability under the scenario of mission independence, the relationship among reliability, maintainability and mission availability can be obtained only with a deformation of (2):
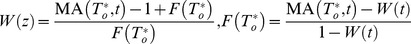
(13)Clearly, if any two of the three reliability measures are obtained, the third one is easy to be calculated. The relationship among system reliability, maintainability and mission availability is displayed in Figure 5 (a). The highlighted curves are the equal-availability curves with the availability values 0.35(0.1)0.95.

The mission availability under the scenario of mission dependence can also be analyzed in the same way. We only give out a numerical example with exponential failure and repair distribution here, while other failure and repair distribution can also be simulated similarly. The relationship among system reliability, maintainability and mission availability is displayed in Figure 5 (b) with exponential reliability rate 0.01(0.01)0.1 and Exponential repair rate 0.002(0.002)0.02.

**Figure 6:**
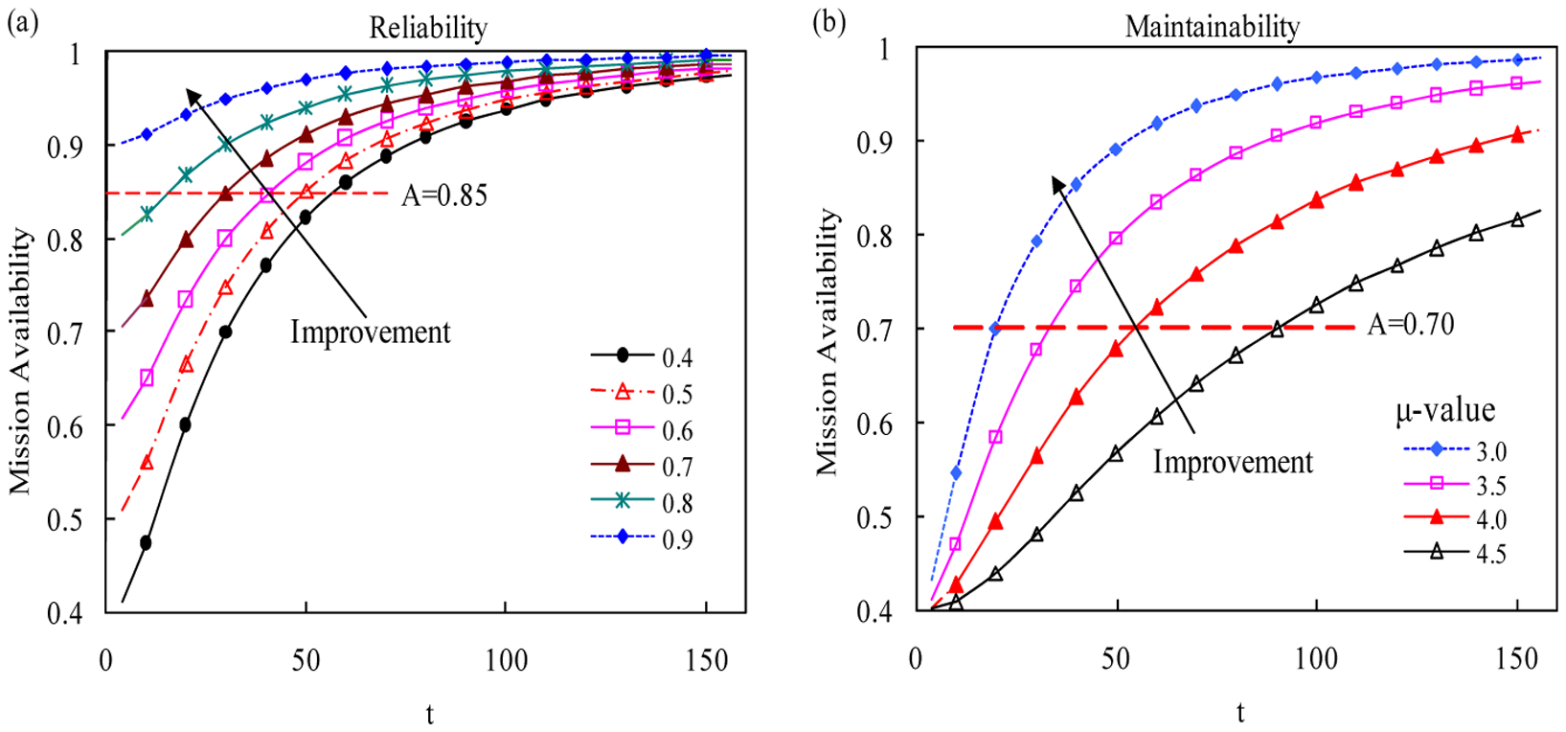
Mission availability trend under different reliability/Maintainability level. (a) Mission availability trend when the system reliability is 0.4(0.1)0.9 and the cumulative repair function is Lognormal distribution with 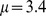, 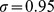. (b) Mission availability trend when the system reliability is 0.4 and the cumulative repair function is Lognormal distribution with 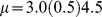, 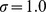. The abscissas of the nodes between the red dotted line and curves represent the least bounded cumulative downtime when the required mission availability is 0.85 in (a) and 0.70 in (b).

Lognormal model is a well-known model for modeling maintainability. So, two special numerical cases with lognormal model are presented as follows. The numerical results are displayed in Figure 6.

In order to investigate the relationship with system reliability, six different values of 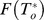 have been examined (namely: 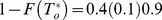), where 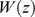 is Lognormal distribution with 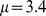, 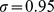. Figure 6 shows that, with the increasing of bounded cumulative downtime, the system mission availability will increase when 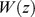 is given. If the bounded cumulative downtime is long enough, the mission availability is approximate to 1. In addition, the reliability level is higher, the time that system mission availability increase to a certain value is less. Furthermore, we set the 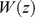 as Lognormal model with 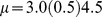, 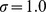 to analyze the system mission availability, where 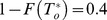. The system mission availability increases with the increasing of bounded cumulative downtime. The mission availability is approximate to 1 when the bounded cumulative downtime is long enough.

### Design and optimum analysis

**Figure 7:**
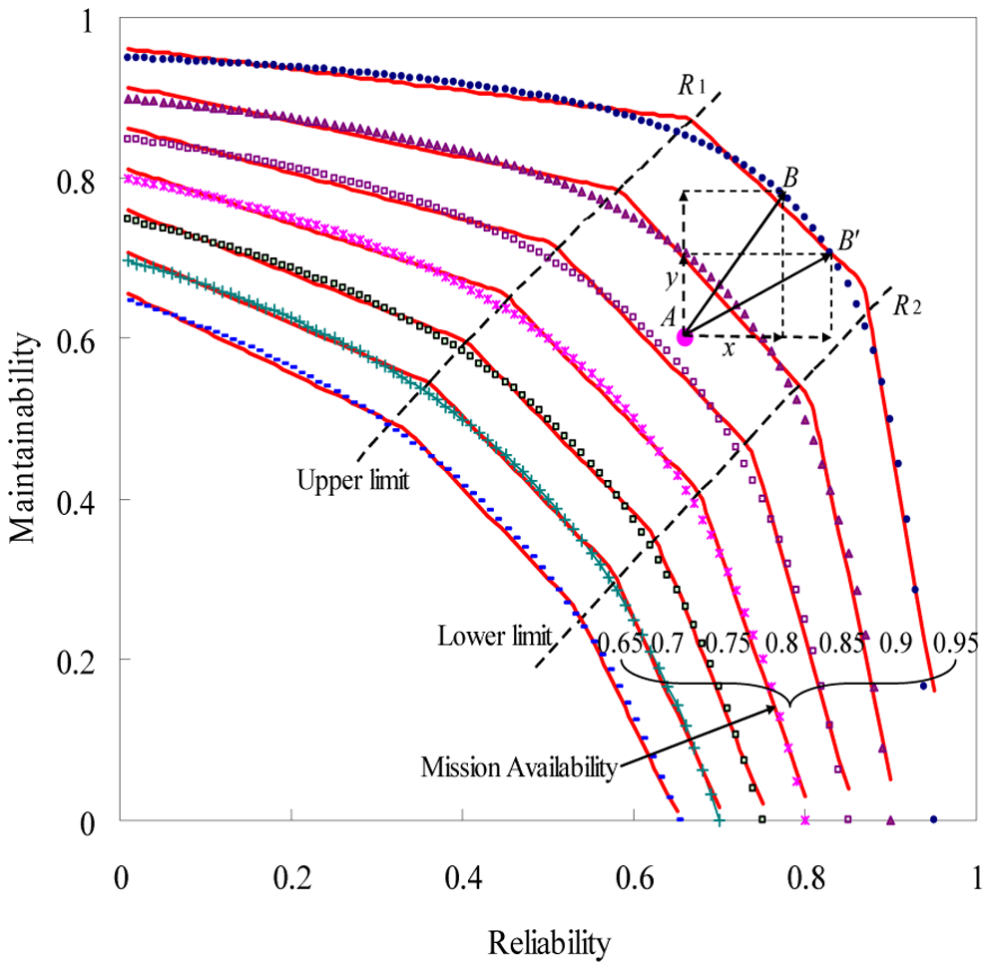
Determination of the no saturation interval. The solid line is the fitted piecewise-linear models. The dashed curve is the change trend between reliability and maintainability with mission availability 0.65(0.05)0.95. The dotted line is the fitted linear models.

How to determine the optimal levels of reliability and maintainability is a very interesting optimization problem when the mission availability is set as a constraint. The optimization problems exist widely in system reliability design and reliability improvement. Figure 7 shows the change trend of reliability and maintainability when the system mission availability is set as a constraint. With the increasing of system reliability, the system maintainability reduces very small before the system reliability reaches a certain value R_1_. On the contrary, the system maintainability reduces rapidly after the system reliability reaches another certain value R_2_. The interval (0, R_1_) and (R_2_, 1] is the saturation interval. And the interval [R_1_, R_2_] is no saturation interval. This phenomenon is defined as saturation effect [30]. The linear approximation [31] is used to determine the no saturation interval. Firstly, we use the piecewise-linear model [31], [32] to determine the change point on each mission availability curve. Then, the change points can be fitted by a certain model, such as the linear model. The nodes of the fitted model and the mission availability curve are the saturation point. So we obtained the upper limit point R_1_ and lower limit point R_2_ as:
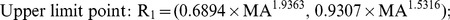


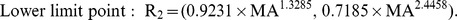



System performance and the cost are acceptable only when the optimal value of reliability and maintainability fall in the no saturation interval. In order to achieve the optimal, we need to trade-off the maintainability and reliability design according to the total cost when the system mission availability is set as a constraint.

Assume the design cost can be presented by the function of maintainability and reliability. The optimal design value can be calculated according to the trading-off cost. Set the current system mission availability level as 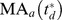, denoted as MA in Figure 7, where the expected system mission availability level is B. We can adopt the method of increasing system reliability or maintainability to satisfy the required system mission availability. In this case, it is important to place the economic analysis to determine the design or improvement level of reliability and maintainability. Set 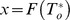 and 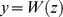, where 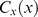 and 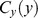 are the cost of 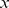 and 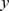 respectively. Then, the optimal reliability and maintainability level can be determined by minimizing the total cost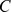:
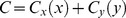
(14)Where 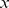 and 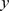 must satisfy:
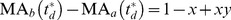
(15)


The optimal reliability and maintainability level can be determined when 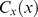, 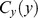, 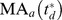 and 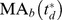 are given. Suppose we have:
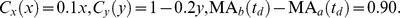
The optimal design or improvement values are 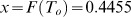 and 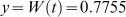
with the minimum cost 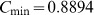.

### Mission scheduling

Consider a system executing production missions circularly. System reliability level is fixed. So the required mission availability should be reached by reason of the unlimited increasing of cumulative downtime [33]. Taking Figure 6 as example, if the system mission availability is required to reach 0.85 and the reliability is 0.4, 0.5, 0.6, 0.7 and 0.8, the required bounded downtime cannot be less than 58, 50, 41, 31 and 16 units of time respectively. More complex, if the mission availability also needs to be optimized, the cost or other criterion can be considered. Here, cost is an optimization criterion.

Assuming the profit of a system production mission performed successfully is *C_p_*, if the system is unavailable for a mission, so the system will lose the profit of *C_p_*. And the expected unavailability cost *C*_1_ can be computed by:
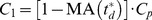
(16)where 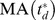 is the system mission availability.

In addition to the unavailability cost, the cost of downtime also affects the profit. Assuming the cost of downtime increases linearly with the increasing of cumulative downtime, we have:
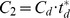
(17)where 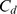 is the expected downtime cost per unit of time. Thus, the total cost is:
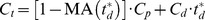
(18)


The mission availability is an increasing function to the bounded cumulative downtime, so the first term of the right side in (18) decreases with 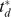 while the second term obviously will be increasing to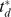. Hence, in order to minimize the total cost of (18), 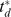
should be optimally selected.

## Limitations of the Study, Open Questions, and Future Work

The aim of this research effort is to present an approximate method to model mission availability for bounded-cumulative-downtime system. Although we have obtained an ideal result, the current study still has some limitations. First of all, we assume the repair is perfect and the system can be restored to the state as new. However, the repair of repairable system may be imperfect. Meanwhile, the mean time between failures will reduce with the usage increasing, while the cumulative downtime will increase. So the cumulative downtimes are not independent and identically distributed. Hence, the traditional reliability models cannot be used to model the cumulative downtimes. The existing repairable system reliability models should be combined in the proposed mission availability model. More research is needed to expand the application scope of the proposed model.

Secondly, the structure dependence and importance of component are not considered in the proposed model. The failure uptimes and downtimes are used to measure the system reliability and maintainability. However, components have different importance and impact on system performance. Hence, the modeling of uptimes and downtimes under considering the structure dependence and importance can be paid more attention to in the further research.

Thirdly, System reliability and maintainability can be obtained by fitting the field failure and repair data. For a new system or with a short operation history, the field failure and repair data may be insufficient to support the modeling accuracy. In the numerical simulations, the maximum percent error in the mission availability estimate is as high as 1.46. Although this implies that there is no noticeable impact on the actual mission availability, more research is needed to study the situation of small sample data to obtain more accuracy results.

Finally, In addition, the proposed model may have some other potential applications. For example, reliability and maintainability allocation, determining the improvement indirect and target at the reliability improvement, maintenance resources optimization and scheduled maintenance. These potential applications will be further researched in the future work.

## Conclusion

In this research, an approximate method was used to model mission availability for bounded-cumulative-downtime system. All failures in a single mission are assumed as one total failure, whose cumulative downtime is equal to the sum of all failures' downtime. The approximate distribution was determined and used to develop the proposed mission availability model. In proposed model, the cumulative downtime and cumulative uptime are set as constrains simultaneously. Then numerical simulations are presented to illustrate the rationality of the assumption and the effectiveness of the proposed model. Finally, the maximum percent error in the mission availability estimate is 1.46. This implies that there is no noticeable impact on the actual mission availability. Based on the acceptable relative errors, the proposed mission availability model is effective and the assumption that takes the cumulative downtime as a variable to model the mission availability is rational.

Due to the closed expression, the proposed mission availability model can be widely adopted. Three important applications were discussed. We have also carried out numerical examples to illustrate the application process. For mission availability analysis, the relationship among reliability, maintainability and mission availability can be obtained. In addition to the design and optimum analysis, no-saturation interval is given out. And a method of determining the optimal reliability and maintainability level is proposed. A method to determine the optimal cumulative downtime with minimizing cost is also suggested for the mission scheduling.
